# Supplementary material for: Planning and optimising CHAT&PLAN: A conversation-based intervention to promote person-centred care for older people living with multimorbidity
Source: PLoS One. 2020 Oct 16;15(10):e0240516. doi: 10.1371/journal.pone.0240516 (PMC7567392; doi:10.1371/journal.pone.0240516)
Supplement: S1 File — (DOCX) [file pone.0240516.s001.docx]

# MOCs interview schedule

## **Background Information for the Interviewer**

- The interview will be semi-structured.
- The exact wording and prompts used may change to encourage the caregiver to share their views. This approach will allow them to discuss their views about their experience at length.

## **Introduction Procedure with caregiver**

- Introduce yourself to the participant and thank them.
  - *Thank you very much for agreeing to take part in the study*
  - Give complete name. Identify self as a researcher from the CLARHC Wessex, Health Sciences, University of Southampton.
- Check that they are still happy to proceed with the interview
- Before recording: *Just have a few things to go through before we start…*
  - *Most interviews last between 60-90 minutes, depending on how much you have to say.*
  - *There are no right or wrong answers, so please say any thoughts, which spring to mind, even if you think they might not be important.*
    - *Also there may be a few long pauses through our conversation where no one is talking, it might seem quite unnatural but if it happens there’s nothing to worry about, the gaps are just to give you time to think about anything else you want to say and also what my next question might be.*
  - *If you are happy, we will record the interview, so we can listen again to what is being said.*
    - *No one will be able to identify you from what you say and everything you tell me will be treated in the strictest confidence. We will not keep anything on record that identifies you, or where you live or anyone else that you mention, so it will all be anonymous.*
    - *We can take a break at any time you like, please just let know and I can pause the recording. We can also stop the interview at any time if you want to.*
  - *Do you have any questions before we start? If you have any questions during the interview I can answer them at the end once we’ve finished recording.*

- Notify participant you will start recording
- Remind them about the purpose of the interview (to find out how living with multiple conditions after cancer and to hear their views on the CHAT and PLAN protocol)
- Ask if they have any questions.
  - *Today the interview will be split into two parts. I will explain each part before we begin it.*

**INTERVIEW: PART 1**

### **Aim**

To understand current practice in supporting caregivers of older cancer patients with multimorbidity to manage their health and meet their health-related goals, with a focus on treatment burden and patient capacity

### **Questions**

The initial questions will explore how caregivers care for those living after cancer in the context of aging and preexisting multimorbid conditions. The interviewer will therefore explore the following general areas in an open fashion. The primary questions are numbered and highlighted in bold font, with further prompts presented as sub-points.

| **The aim of this part of the interview is to understand what it is like caring for someone who has had cancer and also has other conditions. I just want to find out your views on how you support______ (name/ your partner/parent etc) to live and manage these conditions and treatments.We want to find out not only how the illness affects how you care for them, but also what you think about how the conditions are managed. For example, do the medications or appointments affect their everyday life or the role you play as caregiver, and if so in what ways? We want to know more about what works well and what works less well for you both.****Can you talk me through what a typical day looks like for you?** |
| --- |
| - 1. How do you help *______ (name/ your partner/parent etc) to* manage their conditions**? (**activities/work) |
| - 1. What influences makes it easier or more difficult? |
| - 1. What strategies do you have/ What do you do to help them to cope with their conditions/symptoms? |
| 1. **What is a bad day like?** *(rephrase as needed to embed into conversation)* |
| - 1. What makes it a bad day? What works less well on a bad day? |
| - 1. What/does anything make it more difficult to help *______ (name/ your partner/parent etc) to* manage their health/ symptoms? |
| - 1. What part of helping them to manage their health takes up the most time/ energy/ attention? |
| - 1. What part is most challenging? |
| - 1. What might make things easier on a day like this? |
| **What is a good day like?** *(rephrase as needed to embed into conversation)* |
| - 1. What makes it a good day? What works well on a good day? |
| - 1. What would make it easier to have more of these good days? /help you both to have more good days? |
| - 1. What/ does anything make it easier to help *______ (name/ your partner/parent etc) to* manage their health/ symptoms? (*essentially, can you reduce work/ bolstering capacity)?* |
| 1. **Does anyone help you in providing support for______ (name/ your partner/parent etc)?** |
| - 1. In what way? How often? |
| - 1. Do you face any challenges in accessing support? / Have you faced challenges in accessing support and have they been addressed and resolved now? |
| - 1. Do you get support from anyone else? |
|  |
| 1. **How does having more than one condition affect your ability to help** *______ (name/ your partner/parent etc)***?** |
| 1. **How does caring for** *______ (name/ your partner/parent etc)*  **impact on your day-to-day life?** |
| - 1. Does it fit in easily?   2. Does it stop you from doing anything?   3. Has it brought anything positive to life, such as connections with others, knowledge, purpose? |

## **INTERVIEW: PART 2 Questions to ask about the CHAT and PLAN protocol**

### **Aim**

To elicit caregiver views and experiences of the prototype intervention materials will provide insight into whether intervention materials are acceptable, interesting, persuasive, easy to use, and feasible for people to adhere to

### **Questions**

| **This is a study to develop a way to help people to have a useful conversation with their healthcare provider. In this second part of the interview, I will ask you to tell me about what you think the plan we have come up with and what we could do to make it better.**Views on intervention potential. |
| --- |
| - - How would you feel about *______ (name/ your partner/parent etc)* having this sort of a conversation about their health? |
| - - Do you think it could be useful to have a conversation like this? Which part of the conversation would you find most useful and why? |
| - - Could you tell me about how this might work in practice? |
| - - What role might a caregiver play in this? |
| - - Can you tell me about any advantages? |
| - - Can you think of any disadvantages there might be? |
| What did you think of the CHAT and PLAN protocol overall? |
| - - Can you tell me about anything that you liked about the CHAT and PLAN protocol? |
| - - Can you tell me about anything that you disliked? |
| - - Can you tell me about anything that you would change? |
| - - Can you tell me about anything that you thought was particularly relevant to you? |
| - - Can you tell me about anything that didn’t make sense? |
| - - How do you think that should be changed? |
| Questions about the procedures of the trial (format, content, mode, timing and delivery of support) |
| **We are trying to find out a little more about who might be best placed to offer support like this.** |
| - What skills would a healthcare or social worker need to have to lead a conversation like this? What training should be involved for the individual who delivers the intervention? |
| - How should this be delivered? Face-to-face/ telephone sessions? |
| - How many sessions would be needed? How often? |
| - Where could this conversation take place? At patients home? In clinic? |
| - Over what period of time? (Post-cancer treatment? Or Coming to the end of treatment?) |
| **Other Prompts** |
| - *Can you tell me a bit about that?* |
| - *What would you think about that?* |
| - *Can you tell me a bit more about why you think that?* |
| - *What is it you like about that?* |
| - *How did you find that?* |
| - *What was it like?* |
| - *What about that did (not) appeal to you?* |
| - *That’s really interesting* |
| **At close of Interview…** |
| The interviewer will ask the participant if there are any issues they would like to mention which haven’t been covered. And thank the participant, and reiterate that all they have discussed is confidential. |

## Topics of interest to the researcher

| **Successful self-management** | - Greater capacity to continue onward, to reframe their lives, tap into their resources, engage their social networks, and fit their treatment into daily routines - Furthered ability to access and use healthcare, adapt, and self-manage. |  |
| --- | --- | --- |
| **Focus questions on:** | | |
| Patient workload | - **What activities/work patients have to undertake to help them manage their conditions** - Demands include: the resources and limitations affecting patients' ability or readiness to do work, including job, family, travel/transportation, childcare, scheduling and attending clinical appointments, preventive care, self-education, self-care, taking medications, health behaviors, caregiving, and paperwork |  |
| Patient capacity | - What capacity patients have to help them manage their conditions - Patient capacity: the resources and limitations affecting patients' ability or readiness to do work |  |
| **Probe about: Thoughts/ ideas about their treatments and management** | | |
| 1. What they currently do to stay healthy. | - Individuals’ own priorities. - Self-management goals |  |
| 1. What is consuming patients' time, energy, and attention? | - Worries, side effects, appointments, adherence - How many different types of clinics or therapists they need to attend and how they manage this? For example, how often do they go to the GP or hospital each week or month? - Access to health care? How difficult or easy it is to get appointments, access advice? - Participant’s confidence in their health professionals? - Co-ordination of care and adequacy of communication between professionals and professionals and themselves? |  |
| 1. What influences their ability to manage demands? | - How does their workload serve to limit or grow their capacity? - How do patients reduce workload and/or access support (essentially, bolstering capacity)? |  |
| **What BREWS patient capacity? [1]** | | |
| Biography | - Bothersome symptoms that disrupt normal life? - Ability to make meaning in their lives alongside their chronic conditions - Their illness and its required treatments - Coping emotionally - Ability to cope? Feeling of control over the situation? Acceptance or resentment of their conditions? |  |
| Resources | - Patients ability to mobilize new and existing resources in order to access and use healthcare or enact self-care. - Time - Literacy   - Health literacy, from practical experience - Physical abilities and self-efficacy. - Knowledge   - Who do patients discuss their health with? Discuss concerns or review their management plans?   - Lifestyle or other advice? From whom? What is their understanding of this advice?   - How else they have found out about treatments and management of their conditions. (Have they asked friends and family, read leaflets or books, watched television, looked on the internet.) - Have they had to get any adaptations for their home (such as a chair lift or rails), or aids to get out of the house (such as a stick)? - Transportation - Do they face any challenges in accessing social services support? - How participants organise getting prescriptions/ to appointments - Financial implications?   - Access to paid supportive services |  |
| Work (realization of) | - What strategies, if any, participants adopt to improve their wellbeing and self-management of their conditions?   - Normalize work or make it routine.   - Prioritize their conditions or alternatively synchronize demands (based on time or other constraints)?   - Competing life priorities, competing conditions, overwhelming treatment burden, and complex healthcare environments   - Manage expected life roles, leisure activities, and cultural norms.   - Prioritize across conditions   - Navigate the healthcare system   - Organise or coordinate resources, limitations, and their environments while coping with these demands - How, if at all, have they modified daily and social activities - Does healthcare fit the patient’s life or interfere with competing priorities in life? |  |
| Social | - Personal ability to socialize/ compromised ability? - The ability of their social network to accept the patient’s chronic condition(s) and the changes the condition(s) had caused   - How friends and family feel about their illnesses and treatments?   - Do they ask friends and family to help with treatments and lifestyle changes or instead do everything themselves? - How does it affect their life at home, leisure and social activities? - Provision of instrumental support - Social relationships with their healthcare teams.   - Kindness, empathy,   - Fit of the condition, self-care, and treatment/ healthcare plan - Social isolation |  |
